# Utilizing PRISM and RE-AIM to implement and evaluate the Rural Telementoring Training Center (RTTC) for health care workforce development in rural communities

**DOI:** 10.3389/frhs.2023.1219308

**Published:** 2023-10-19

**Authors:** Trisha V. Melhado, Suyen Schneegans, Andrea Rochat, Keito Kawasaki, Erin P. Finley, Denna Wheeler, Waridibo E. Allison

**Affiliations:** ^1^ Center for Health Policy, Division of Academic Innovation, University of North Texas Health Science Center, Fort Worth, TX, United States; ^2^ Center for Research to Advance Community Health (ReACH), The University of Texas Health Science Center at San Antonio, San Antonio, TX, United States; ^3^ Center for Rural Health, Oklahoma State University Center for Health Sciences, Tulsa, OK, United States

**Keywords:** telementoring, Rural Telementoring Training Center, workforce development, rural, health

## Abstract

**Introduction:**

Amid rural health worker shortages and hospital closures, it is imperative to build and maintain the local workforce. Telementoring (TM) or technology-enabled mentoring, is a tool for improving health care quality and access by increasing workforce capacity and support. The national Rural Telementoring Training Center (RTTC) was developed to compile and disseminate TM best practices by delivering free training, tools, and technical assistance to support the implementation, sustainability, and evaluation of new and current TM programs for rural health workers. This paper details how the Practical, Robust Implementation and Sustainability Model (PRISM) was used to understand the context that shaped implementation as well as how Reach, Effectiveness, Adoption, Implementation, and Maintenance (RE-AIM) was concurrently applied to frame outcomes.

**Methods:**

The RTTC has three implementation strategies: outreach, training and technical assistance (TTA), and a Quality Measure Toolkit. Ongoing periodic reflections with the RTTC team, informed by PRISM, were collected, as were RE-AIM outcomes. Central to this design was the continuous review of incoming data in team meetings to inform programmatic changes by identifying challenges and applying modifications to strategies in real time.

**Results:**

Major implementation changes discussed during reflections included providing timely and relevant messaging through various platforms, streamlining and customizing a TTA approach, and offering different options for accessing the Toolkit. The outreach strategy resulted in high Reach across the US, with over 300 organizations contacted. The effectiveness of the RTTC was demonstrated by counts of people engaging with outreach (ex. over 8,300 impressions on LinkedIn), the website (over 6,400 views), and e-bursts (33% open rate). Moreover, there were 32 TTA requests and 70 people accessing the Toolkit. Adoption was demonstrated by 27 people participating in TTA and 14 individuals utilizing the Toolkit.

**Discussion:**

The integration of PRISM and RE-AIM frameworks promoted a holistic implementation and evaluation plan. Using PRISM, the RTTC team was able to reflect on the implementation strategies through the lens of contextual factors and make rapid programmatic changes within team meetings. That process resulted in outcomes framed by RE-AIM. The integration of two frameworks in tandem provided an adaptive and comprehensive approach to implementing a large-scale, national program.

## Introduction

Approximately 15% of Americans (46 million people) reside in rural areas (1, 2). Many residents in rural and remote communities do not have regular access to comprehensive and affordable care due to a scarcity of services, hospital closures, and a shortage of primary care providers and specialists (3, 4). A recent report from the federal Health Resources and Services Administration (HRSA) showed that 65.6% of Primary Care Health Professional Shortage Areas (HPSAs) are located in rural areas (5). Roughly 136 rural hospitals have closed from 2010 to 2021; additionally, more than 600 additional hospitals, representing about 30% of all rural hospitals in the country, are at risk of closing (6, 7). Even though access to quality and affordable health care does not guarantee better health outcomes, it is necessary for promoting and maintaining health and well-being (5). With rural health care worker shortages and hospital closures, it is imperative to build and maintain the workforce in rural settings.

Telementoring (TM), or technology-enabled mentoring, is the use of telecommunication technology to deliver training, education, and support that builds health care capacity. As a highly adaptable tool for connecting learners regardless of their geographic spread, TM allows organizations to build capacity by sharing evidence-based curricula, supporting delivery of practice-based, culturally responsive care, advancing the skillsets of health care workers, promoting task-shifting to increase access to specialized care, and creating virtual communities of practice and learning to empower the workforce (8). Despite the new and promising strategies TM offers for providing remote training and improving care access, there is not a solid evidence base for implementation strategies or common data elements for evaluating program quality (9).

To address this, HRSA funded the Rural Telementoring Training Center (RTTC), a national program to promote and support delivery of high-quality TM programming that increases rural workforce capacity and improves rural health. The RTTC was developed with the goal to compile and disseminate TM best practices by delivering free training, tools, and technical assistance to support the implementation, sustainability, and evaluation of new and current TM programs for rural health workers. Notably, the RTTC does not run TM programs on specific topics—it engages with and supports organizations in designing, implementing, and evaluating their own programs in response to local community priorities. This train-the-trainer approach, which focuses on building capacity and sharing tools to help others lead, was established to promote local uptake of TM, responsiveness to community needs, and program sustainability. Finally, to build awareness about the value and breadth of TM, the RTTC was designed to be a source of open information and widely share expertise for a range of rural health stakeholders, such as policymakers, health workers, community organizations, and the general public.

The implementation and evaluation of the RTTC are guided by the Practical, Robust, Implementation and Sustainability Model (PRISM) and the Reach, Effectiveness, Adoption, Implementation and Maintenance (RE-AIM) implementation science frameworks (10, 11). These frameworks were selected because they provide guidance for the successful execution and long-term sustainability of the RTTC’s interventions and serve as a comprehensive evaluation tool for the program. As a conceptual model guiding implementation, PRISM is used to identify key contextual factors at various levels of organizational structures (e.g., external and internal contexts) that impact program implementation, evaluation, and sustainability (10). RE-AIM provides a framework for evaluating program outcomes (10). PRISM and RE-AIM are often used together to ensure coordination between a program’s implementation and evaluation efforts (12–14). With the RTTC being a novel program, it was important to establish a framework that offered a comprehensive approach to understanding the context behind implementation changes and guided evaluation. The iterative use of RE-AIM has been previously noted (15, 16). This paper details how PRISM was used to understand the context that shaped RTTC implementation strategies through qualitative assessment, as well as how RE-AIM was applied to frame quantitative outcomes during the first two years of implementation.

## Materials and methods

### Intervention

The RTTC is a novel program that helps organizations design, implement, and evaluate TM programs to train and support rural health workers. A preliminary step in the establishing the RTTC was to promote it across the United States to increase visibility and recruit organizations (i.e., “Learning Partners” or LPs) who have interest in establishing TM for rural health workers. Concurrent with this was the development of a protocolized structure for delivering Training and Technical Assistance (TTA) to support LPs throughout the continuum of implementation—from program design to scaling and evaluation. The RTTC focuses on six TM models: Adapted Community Health Clubs (ACHC), Extension for Community Healthcare Outcomes (ECHO) (17), individual consultations, online modules and curricula, podcasts, and webinars. Table 1 summarizes descriptions of the models, including their main elements and the value propositions for each model. This information is shared with LPs as they consider which TM model to implement and/or evaluate. The RTTC supports LPs in identifying topic areas and a target audience based on their unique context.

**Table 1:** Telementoring model descriptions, key content, and value proposition.

Model descriptions	Key content	Value proposition
**Adapted community health club:** groups of health workers who gather virtually for regular, structured sessions to support each other, learn about a health topic, and organize health action	• Didactic presentations • Interactive discussions and activities	• Peer community and support • Collaborative learning • Sustainable behavioral change and community-led action • Adaptable to regional and cultural differences
**ECHO:** Hub-and-spoke model using videoconferencing to connect a team of subject-matter experts with community-based individuals	• Didactic presentations • Deidentified case presentations • Interactive non-hierarchical discussions	• Interactive peer to peer-learning • Establishes communities of practice and learning • Highly adaptable • Expansive network of ECHO hubs for collaboration
**Individual consultation:** Structured one-on-one interaction through telephone or video- conference between a specialist and health care worker	• Established workflow for consultation • Structured case forms • Synchronous or asynchronous • Post-consultation documentation	• Efficient, direct, and documented communication • Increases access to specialty knowledge due to rapid consultation turnaround • One on one nature allows for direct relationship building
**Online modules & curricula:** Self-paced learning via online modules and slides with or without audio	• Learning management system content • Course syllabus and other resources • Asynchronous communication with trainers may be possible	• Personalized educational experience compatible with schedules • Potential for online communities centered around the training content • Evergreen materials with updates only as practice and standards change
**Podcasts:** Audio (or audio with visual enhancement) broadcasts	• Thematic episodes or series • Content delivered through array of formats • Available for download on a computer or mobile device	• Engaging format • Flexibility with access and adaptiveness to learner interests and priorities • High quality content with relatively low-cost expenditure
**Webinars:** Live audiovisual presentations delivered by an individual or panel with a discussion and interactive question/answer component	• Content delivery to an audience of any size via online platform • Live interaction possible (Q&A session or chat feature) • Single topic or series	• Rapid launch possible • Can deliver high dose of educational content • Can be recorded and archived for later distribution and audience use

### Implementation strategies

Three strategies guided RTTC implementation. First, we developed an overarching outreach strategy, which included two national events, an RTTC Launch Week and a virtual UnConference (18). Second, we established a TTA pathway, which included a piloting phase as well as development of an LP needs assessment, structured workflow, and a custom learning management system with resources on TM models. Third, we created and disseminated a Quality Measure Toolkit to describe various TM models, evaluation activities, and the TTA process, in order to support LPs in the continuous quality improvement of their TM program. In developing these implementation strategies, the RTTC team integrated PRISM constructs of sustainability, organizational/staff, implementation, recipient, and external context. Specifically, strategies focused on supporting long-term sustainability of services and assessing RTTC staffing to efficiently support implementation. Tools and resources developed during the implementation timeframe were assessed and modified to best reach the recipient audience (existing and potential LPs). All strategies were developed with sensitivity towards recipients’ priorities and an awareness of how the external context (e.g., local and national events) might influence program activities.

The comprehensive outreach strategy developed for the RTTC included social media accounts for LinkedIn and Twitter, e-mail marketing (e-bursts), a website, conference presentations and exhibits, invited presentations, virtual meetings with potential LPs, and creation of a portfolio of digital and hard-copy knowledge-dissemination materials. The goal of the outreach strategy was to increase RTTC visibility, spread understanding of TM, and engage LPs. Given such an outward-facing goal, it was important to ensure consistent messaging and branding across the Center; to that end, a style guide was developed along with programmatic talking points. Social media posts on Twitter and LinkedIn occurred on average biweekly. E-bursts were sent bimonthly through Nutshell, a customer relations management (CRM) system traditionally used for sales, which the RTTC adapted as a tool for organizing and tracking interactions with LPs and its national audience (19). The website, social media, and e-burst platforms each offered native analytics tools to identify audience engagement and performance (e.g., impressions, link clicks, open rates). Presentations included panel and individual talks, while conference exhibits targeted rural health audiences. Outreach meetings allowed potential LPs to interact directly with the RTTC team. The library of knowledge dissemination products included informational flyers and descriptions of TM models. The RTTC hosted two virtual outreach events to increase Center visibility, establish connections in the rural health community, and recruit LPs. RTTC Launch Week, held in the second year of implementation, showcased the release of the RTTC’s online catalogue of courses to introduce learners to an array of TM models. The event additionally featured invited speaker presentations and a panel discussion on topics relevant to telementoring and rural health care. During the third year of program implementation, the RTTC hosted a virtual Rural Telementoring UnConference, which leveraged a destructured conference format (20) to create space for incubating ideas and building partnerships around salient topics in rural health.

Piloting TM models helped the team refine its TTA process and resources by allowing them to work closely with implementing partners as they developed their programming. Piloting partners were identified prior to funding from within the grantee’s organizational network, with a focus on recruiting across multiple models and implementation stages. Through regular meetings, the TTA team learned about the organizations, their topic of interest, target learners, and which TM model they were interested in piloting. Three pilots were initiated: a podcast on HIV prevention and treatment, a webinar on e-cigarette use for an oral health practitioner network, and an ACHC to recruit and train community health workers within five South Texas Area Health Education Centers (AHECs). Throughout these pilots, the TTA team gained experience in delivering an array consultative guidance on key implementation steps such as identifying a target audience, establishing curricular schedules and content, developing marketing and communication strategies and materials, and acquiring and training in appropriate hardware/software. This process provided insight into the resources organizations might need to implement and evaluate TM as well as strategies for delivering individualized, structured TTA to meet the unique needs of future LPs. Resources developed as a result of this were: (1) a needs and readiness assessment tool to understand LPs’ organizational capacity for delivering TM; (2) a structured TTA workflow to assist LPs at any stage of implementation; and (3) a customized Learning Management System (LMS) containing introductory curricula for each TM model (21). Further guidance on structuring TTA was gained from established TTA manuals (22).

A Telementoring Quality Measure Toolkit was developed and implemented as a strategy to ascertain quality measures for different TM models and provide a mechanism for their evaluation (23). The Toolkit offers quality measure checklists for five TM models and recommendations for how users can assess their program and identify areas for improvement across an array of domains: Staffing, content, technology, learner, marketing, sustainability, instructor, and impact.

### Data collection

**Figure 1:**
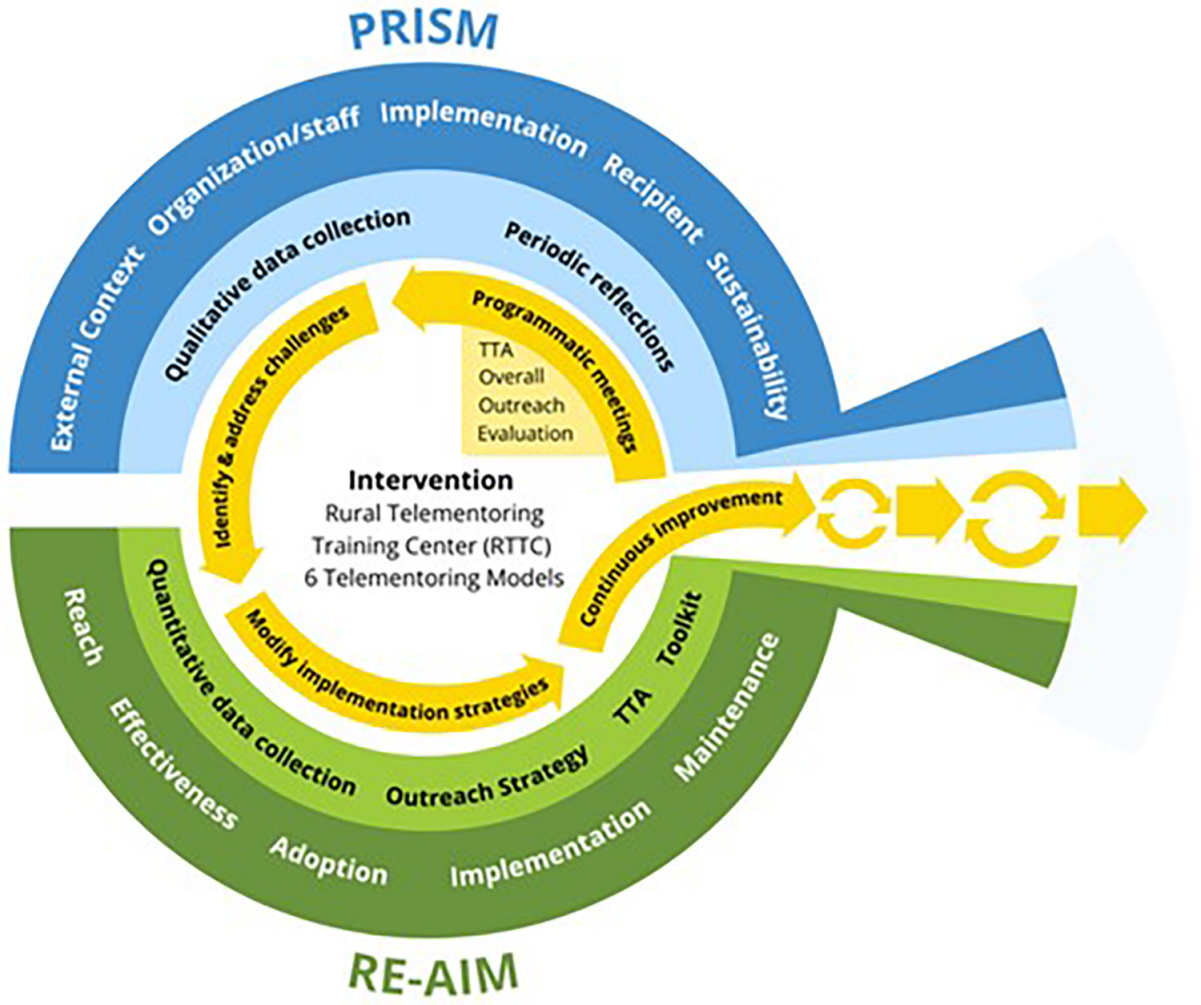
RE-AIM/PRISM model adapted for RTTC. Adapted from: Reed et al. (25).

Mixed-method evaluation of the RTTC occurred from September 1, 2021 through March 31, 2023 using the PRISM and RE-AIM frameworks. Theory-informed evaluation data were collected (including periodic reflections, data on outreach, TTA, and the Toolkit, etc.) and continuously reviewed in team meetings. The PRISM framework helped the RTTC team attend to factors relevant for program implementation (24). Figure 1 demonstrates the interplay of PRISM and RE-AIM and how they provided conceptual scaffolding for the RTTC (25). Periodic reflections, informed by PRISM, were collected as an ongoing evaluation activity, as were RE-AIM outcomes. Central to this design was the continuous review of incoming data in team meetings to inform larger programmatic changes by identifying challenges and modifying the implementation strategies. Periodic reflections then provided opportunities for discussing trends on a longer time scale (quarterly), as well as reflecting on- and learning from programmatic shifts over time.

### Qualitative data gathering

Periodic reflections (reflections) are a semi-structured qualitative methodology that apply ethnographic principles to track internal and external factors impacting program implementation in real time (26). Aligning with PRISM, periodic reflections allowed us to examine contextual influences on RTTC implementation (Table 2). The five PRISM constructs are: (1) External context—the broader societal environment; (2) Organization/ staff—the team structure of the RTTC; (3) Implementation—strategies for designing and executing the RTTC intervention; (4) Recipient/LP—factors that impact participation in TTA or TM implementation; and (5) Sustainability—factors that ensure long-term implementation of TM. Separate team meetings took place biweekly to discuss implementation strategies while periodic reflections offered a place for the RTTC team to contemplate these through the lens of PRISM constructs. Starting in the first quarter of implementation, RTTC team members—including the primary investigator, project coordinator, research assistant, project administrator, and team leads representing evaluation, TTA, and outreach—participated in monthly reflections. Team members from a partnering organization that helped to facilitate TTA participated in quarterly reflections during the second quarter of implementation; these sessions included a TTA facilitator, curriculum advisor, and assistant. In total ten team members, seven RTTC core team members and three TTA consultant team members, participated in the reflections.

**Table 2:** RTTC periodic guided reflection—PRISM constructs addressed.

Guided reflections components-questions	PRISM constructs addressed
Status Update—What are the current main activities for RTTC? How is it going?	Organizational/staff, implementation, recipient/LP factors, sustainability
Adaptations to Intervention/ Implementation Plan—Have there been any changes to the implementation plan or how the intervention is delivered in the past month?	Organizational/staff, implementation, recipient/LP factors, sustainability
Stakeholder Engagement—Have there been any stakeholder engagement efforts in the past month?	Recipient/LP factors
Environment/Context—Have you seen any recent changes in the local or national environment that you think may have impact for implementation?	External Context
Quick Check—What’s going well right now? What’s not going well right now?	Organizational/staff, implementation, recipient/LP factors
Planning—What are the next steps going forward?	NA

Adapted from: EP Finley, AK Huynh, MM Farmer, B Bean-Mayberry, T Moin, S Oishi, JL Moreau, KE Dyer, AB Hamilton. *EMPOWER QUERI Tip Sheet on Periodic Reflections*. September 2017. September 2017—We gratefully acknowledge funding from VA QUERI (QUE 15–272).

The reflections discussion guide inquired about recent team and LP activities, implementation progress and challenges, changes to the implementation plan, and changes in the national or local environment that might impact implementation and next steps. Follow-up questions and additional probes were utilized to obtain a full understanding of the implementation landscape. The discussion guide was adapted from the reflections template (26) and all reflections were held via Zoom. While implementation changes took place during team meetings, reflections provided an opportunity for the RTTC team to consider the larger program and how changes were influenced by contextual factors. RTTC team reflections were facilitated by faculty with training and expertise. A trained research associate conducted reflections with collaborative partner organizations. All reflections were recorded and/or transcribed with at least two note takers to ensure notes were near-verbatim and comprehensive.

### Quantitative data gathering

Table 3 describes how the RTTC’s quantitative evaluation was structured to align with RE-AIM outcomes. For the current evaluation period, we assessed the RTTC’s reach, adoption, and effectiveness. Reach was demonstrated by recipient counts of the implementation strategies while adoption was demonstrated by use of TTA and the Toolkit. The overall effectiveness was demonstrated by engagement with the implementation strategies. For example, an LP may receive a LinkedIn post about the RTTC (reach), become an LP (adoption), and share the LinkedIn post with colleagues (effectiveness). Conversations around sustainability took place since program start, details of which are part of the reflections results. Implementation and maintenance will be planned in future assessments, whereby feedback will be obtained from recipients of the RTTC implementation strategies to determine whether the strategies were delivered in an effective manner and in accordance with the implementation plan. Plans are underway to assess TTA quality among LPs and Toolkit satisfaction and usability.

**Table 3:** RTTC evaluation—RE-AIM domains and measures by implementation strategy .

RE-AIM domains, measures	Implementation strategy
Reach	
Number of recipients of outreach strategy initiatives	Outreach
Number of contacts with potential Learning Partner (LP) organizations	Outreach/ Training and Technical Assistance (TTA)
Number of people accessing the Telementoring Quality Measure Toolkit	Toolkit
Effectiveness	
Engagement with outreach strategy platforms Launch Week and UnConference attendee characteristics	Outreach
Characteristics of LPs contacting the RTTC for TTA	TTA
Characteristics of people using the Toolkit’s features	Toolkit
Adoption	
Utilization of TTA by LPs	TTA
Use of quality measures checklists	Toolkit
Implementation	
Under development	Outreach/ TTA/ Toolkit
Maintenance	
Under development	Outreach/ TTA/ Toolkit

### Data analysis

Transcripts and recordings from the reflections were reviewed independently by two experienced qualitative researchers using rapid qualitative analysis (27–29). Analysis occurred through several steps: Two researchers independently listened to the recordings, reviewed session notes, identified key findings as they related to PRISM constructs, and populated a data matrix. These data were distilled and refined in the analysis. The rapid analyses allowed for broad understanding of the factors impacting RTTC implementation while execution of implementation strategies was still occurring. The main factors of interest were organized according to the PRISM constructs. Information repeated in multiple reflections was highlighted as a salient finding. This approach allowed for a high-level understanding of the reoccurring contextual factors impacting the implementation strategies. Based on the Framework for Reporting Adaptations and Modifications to Evidence-based Implementation Strategies (FRAME-IS), key adaptations to the implementation strategies discussed during reflections were noted (30). RE-AIM outcomes of reach, adoption, and effectiveness were summarized using descriptive data. Qualitative findings regarding PRISM-related contextual factors and quantitative findings reflecting RE-AIM outcomes were integrated to explore how RTTC implementation evolved as the program matured and how iterative refinement of the program’s outreach, TTA, and Toolkit strategies occurred during this period.

## Results

### PRISM constructs

#### External context

Local and federal governmental leadership, policy changes, and the COVID-19 pandemic—inclusive of Public Health Emergency Declarations—were prominent external factors that influenced RTTC implementation. Given the RTTC’s goal to be a reliable source of information for a wide range of stakeholders, including policymakers and the general public, the team paid close attention to shifts in local and federal government that might influence rural health care delivery and telementoring. Throughout the project, the team noted emerging priorities at the local, state, and federal levels on topics such as broadband access, availability of a community-based health workforce, technology-supported health care delivery (e.g., telemedicine and telehealth), emergency and disaster preparedness, and strategies to address growing rural health disparities. The RTTC’s outreach mechanisms—whether to specific LPs during consultations or to broader audiences through e-bursts, social media posting, or presentations—offered a platform to educate about the shifting landscape of rural health delivery and offer information on how telementoring can be used as a tool to address these priorities. For example, ongoing closures of rural hospitals, which threatened to cut off care access and destabilize local economies, also reinforced the need for leveraging TM to cross-train, task shift, and maintain local care capacity. The external context of the COVID-19 pandemic and ensuing restrictions on in-person events caused the RTTC to pivot its in-person outreach and recruitment strategies to entirely virtual formats.

#### Organizational/staff

The organization of the RTTC’s implementation strategies centered on administrative tasks, such as staffing and establishing roles and responsibilities of team members. Given the national scale of the RTTC and expected staff changes from attrition and new hires, an organizational structure was created consisting of Outreach, TTA, and Evaluation arms to streamline the Center’s activities. Documented roles and responsibilities created a consistent staffing structure to ensure that all arms of the implementation strategy had sufficient personnel to conduct implementation tasks and activities.

#### Implementation

There were several modifications to the original implementation plan. One modification was changing the original geographical target regions for LP recruitment to focus on each of the ten geographically defined HRSA regions (31). This became a new benchmark for measuring the success of the RTTC—reach across all 10 HRSA regions through the implementation strategies. After program launch, the funder (HRSA) provided a list of potential collaborators which guided initial outreach efforts. Another positive modification to the original outreach plan was the development of a comprehensive strategic communication plan informed by training of two faculty team members (32, 33). This strategic communication plan guided internal and external messaging by establishing RTTC branding, messaging, and communication protocols—resources that became necessary to support the Center’s growth. Key components of the strategic communication plan included identifying and understanding target audiences, creating an RTTC branding guide and shared talking points, establishing a crisis communications plan to anticipate and protocolize steps for responding to program risks, and guidance on aligning outreach efforts with program goals.

From the outset, outreach activities and milestones were tracked to assess the impact of the RTTC. Additionally, given the national scope of the RTTC, project management software solutions were utilized to manage large-scale implementation across all arms. Monday, a cloud based project management software, was used to track the overall management of the RTTC including outreach (35), while Nutshell, a customer relations software, and Wufoo, an online form builder that integrates with Nutshell were used to track LP meetings and communication (19, 36). Collectively, the software programs allowed for seamless communication with RTTC team members and LPs.

#### Recipient

A salient theme in how recipients impacted RTTC implementation was the sense of urgency for getting resources, like the TTA workflow and LMS content, ready for LPs. The recipient construct allowed the TTA team to consider how best to address LP challenges, such as staffing shortages, lack of organizational support and funding, understanding of marketing strategies, and knowledge about evaluation, while also building on strengths, such as LPs having a synergistic team, established partnerships, and engagement from administrators. This awareness helped the TTA team tailor consultation for each LP based on their strengths while mitigating implementation barriers. LPs seeking TTA guidance primarily represented state health departments, non-profits, and programs within academia. The TTA process was flexible and fluid to meet unique LP needs. Tools such as the needs and readiness assessment assisted in tailoring the consultation approach as it provided a landscape view of LP’s requests, organizational and financial resources, as well as anticipated goals of their program. Furthermore, TTA meetings were coordinated around LP availability to ensure the process was beneficial and practical.

### Sustainability

It was noted that the materials developed throughout implementation such as the LMS courses, a library of promotional assets, learning kits, and social media accounts factored into the sustainability of the RTTC. All resources developed were intentionally free and publicly available. A Creative Commons license was applied to all deliverables to support their broader uptake and use beyond the funded program period. It was noted that, once established, automated systems such as self-guided LMS courses, would minimize staff burden and promote the sustainability of RTTC resources.

During the reflections, issues regarding the implementation strategies were mentioned. Those discussions led to programmatic refinement during team meetings. Table 4 demonstrates key implementation strategy changes by the challenge identified, data source, adaptation made, and outcome (30). Paying attention to current events related to the rural health workforce ensured that the messaging was relevant while expanding the RTTC presence across several online platforms helped to achieve the goal of targeting messaging to a wide audience. For TTA, the use of software designed for client management was valuable in tracking communications and interactions with LPs—a critical need as the LP pool grew. Lastly, understanding the best way to disseminate the Toolkit was solved by getting LP feedback, which resulted in two different ways to access the Toolkit. Since the implementation strategy data were tracked on a monthly basis, it was easy to see the positive impact of programmatic adjustments in terms of the RE-AIM outcomes. Overall, strategy changes, whether based on external context (i.e., current events), team meetings, or feedback from LPs (i.e., TTA workflow and Toolkit accessibility), resulted in positive outcomes with increased engagement and a streamlined process. The PRISM framework helped the RTTC team structure the Center’s initiatives and identify how the implementation strategies changed through team meetings. The reflections findings showed how the Center’s implementation strategies were adapted, while data from RE-AIM demonstrated the outcomes of such adaptations. In light of that approach, the outcomes were viewed as successful.

**Table 4:** Implementation strategy challenges by data source, adaption, and outcomes.

Implementation strategy	Challenge	Data sources	Adaption	Outcomes
Outreach strategy	Reaching multiple audiences online with relevant content	News feeds specific to the rural health workforce Website, e-burst, and social media metrics	Tracking of current events relevant to RTTC’s target audience Create an array of outreach materials for online use	Relevant messaging resulting in reach and engagement with website, e-bursts, and social media accounts
TTA platform	Tracking and managing communications with LPs	TTA meetings	Utilization of a customer relations management system	Streamlined approach for handling LP communications
Quality Measure Toolkit	Toolkit distribution method	LP feedback	Have multiple ways to access Toolkit	Toolkit platform that allows for real time use and PDF for future use

Adapted from Miller et al. (30).

### RE-AIM domains

#### Reach

The outreach strategy resulted in a high yield of messaging across several platforms to share information about RTTC events and resources. During the implementation timeframe, 383 tweets and 66 LinkedIn posts were created and published on the platforms. The website had over 6,458 visitors, Twitter had a total of 246 followers, and the LinkedIn account had 115 followers. There were 31 presentations, 8 media/policy appearances, and 5 outreach exhibits at national and regional conferences. Thirteen e-bursts resulted in over 4,000 messages sent to the RTTC email listserv. The RTTC contacted 311 organizations across the 10 HRSA regions via e-bursts (Table 5). Overall, seventy unique individuals accessed the Quality Measure Toolkit via the online platform.

**Table 5:** Number of potential learning partner (LP) organizations and actual LPs by HRSA regions.

HRSA regions	Potential LP organizations	Actual LPs
1	10	1
2	13	2
3	20	2
4	42	4
5	25	1
6	128	16
7	16	1
8	25	2
9	9	1
10	23	2
	Total = 311	Total = 32

#### Adoption

Among the 32 potential LPs requesting TTA, 27 LPs met with the TTA team at least once, with 17 LPs having monthly TTA meetings to discuss their program implementation plan. Among the individuals accessing the Quality Measure Toolkit, 14 utilized the checklists and provided quality measure data for their respective TM model. This included data for the following models: ECHO (*n* = 7), individual consultations (*n* = 2), podcasts (*n* = 3), and webinar (*n* = 2).

#### Effectiveness

Outreach activities resulted in nationwide engagement across all 10 HRSA regions with RTTC messaging and demonstrated effectiveness of the outreach strategy. The website had over 16,000 page views by over 5,200 users, with an average session duration of 59 s. The most popular pages, as demonstrated by number of page views, included the Home page (>6,400 views) and Tools and Training page (>1,600 views). Website trends showed peaks in site visits/page views following email campaigns and presentations. Twitter posts received over 125,000 impressions and over 2,600 engagements (e.g., likes, link clicks, profile clicks). LinkedIn posts received over 8,300 impressions, with 561 clicks. Among the e-bursts, there was a 33% open rate. The RTTC Launch Week was attended by 101 people and 107 people attended the Rural Telementoring UnConference from all ten HRSA regions. Among the organizations that have contacted the RTTC, there have been 32 distinct LP TTA requests from 17 states (Table 5). Among the people accessing the Quality Measure Toolkit via an online link, 42 requested a PDF version, 17 chose to go directly to the Toolkit, nine wanted to learn more about the Toolkit’s development, and seven did not choose an option. It was possible for people to access the Toolkit numerous times to choose different options. Table 6 details the HRSA region and attendee characteristics for Launch Week and UnConference attendees, in addition to the characteristics of individuals requesting TTA on behalf of their organization and those accessing the Toolkit.

**Table 6:** Characteristics of launch week and UnConference attendees, TTA requesters, and toolkit accessors.

	Launch week attendees *N* = 101	UnConference attendees *N* = 107	TTA requesters *N* = 32	Toolkit accessors *N* = 70
HRSA Region	*N* (%)	*N* (%)	*N* (%)	*N* (%)
1	6 (6)	5 (5)	1 (3)	0 (0)
2	1 (1)	3 (3)	2 (6)	16 (23)
3	11 (11)	9 (8)	2 (6)	2 (3)
4	14 (14)	9 (8)	4 (13)	6 (9)
5	5 (5)	3 (3)	1 (2)	1 (1)
6	49 (49)	58 (54)	16 (50)	26 (37)
7	4 (4)	4 (4)	1 (3)	3 (4)
8	7 (7)	8 (7)	2 (6)	7 (10)
9	2 (2)	1 (1)	1 (3)	3 (4)
10	1 (1)	7 (7)	2 (6)	5 (7)
Unknown	0 (0)	0 (0)	0 (0)	1 (1)
Profession type				
Academia	0 (0)	5 (5)	8 (25)	0 (0)
Administrator	44 (44)	33 (31)	15 (47)	23 (33)
Analysts	7 (7)	1 (1)	1 (3)	0 (0)
CHW/Social Worker/Health Educator	9 (9)	25 (23)	3 (9)	13 (19)
Coordinators	14 (14)	6 (6)	0 (0)	0 (0)
RN/NP	3 (3)	6 (6)	5 (16)	7 (10)
Pharmacist	1 (1)	0 (0)	0 (0)	0 (0)
Physician	1 (1)	7 (7)	0 (0)	14 (20)
Student	0 (0)	1 (1)	0 (0)	3 (4)
Other	21 (21)	23 (21)	0 (0)	10 (14)
Organization type				
Academic Institution	16 (16)	38 (36)	16 (50)	34 (49)
Government	25 (25)	21 (20)	7 (22)	8 (11)
Health care setting	26 (26)	25 (23)	2 (6)	4 (6)
NGO/NPO	15 (15)	10 (9)	7 (22)	7 (10)
Professional Society	1 (1)	0 (0)	0 (0)	0 (0)
Public health	8 (8)	0 (0)	0 (0)	0 (0)
Other	9 (9)	13 (12)	0 (0)	17 (24)

## Discussion

Utilizing PRISM and RE-AIM frameworks for a comprehensive national program like the RTTC promoted a holistic implementation and evaluation plan. The RTTC team were able to reflect on the implementation strategies through the lens of contextual factors using PRISM constructs while making changes to the strategies within team meetings. Ultimately, that process resulted in outcomes that were framed by RE-AIM. Prior research has used reflections data to retrospectively identify adaptations occurring over the course of implementation (37); this is one of the first studies to explore how reflections can be used to aid teams in identifying challenges in real time, allowing for more timely sense-making, problem solving, and adaptation. This approach allowed for a pragmatic method for integrating reflections as part of operational improvement.

### PRISM and RE-AIM implications.

While previous studies have utilized the PRISM and RE-AIM framework independently, few have integrated them (16, 38). Similar to the handful of studies mentioned by Rabin et al. (38), PRISM helped our team stratify and understand the multilevel contextual factors as they related to RTTC implementation. However, the RTTC stands apart from other comparators in its uniquely broad scope—as a national, disease-agnostic intervention (i.e., training the trainer to conduct telementoring) delivered to recipients who are the convening entities within local contexts, rather than direct users or beneficiaries (i.e., primarily targeting organizations rather than patients or health workers). Use of the frameworks in tandem yielded several lessons learned. While PRISM allowed the RTTC team to consider the context behind implementation decisions, RE-AIM demonstrated quantifiable outcomes from those adjustments. For instance, acknowledging current events related to the rural health workforce allowed the outreach team to create multi-platform messaging that was relevant to potential TM implementers, while engagement data allowed the team to monitor the success of those communications. Or, within the TTA arm, understanding recipient/LP strengths and barriers helped the team customize its consultations. Additionally, the PRISM lens, which emphasizes sustainability, prompted us to make resources freely and publicly available, thereby supporting a long-term pathway for recipients to implement TM. Simply put, consideration of context helped the RTTC provide relevant and sustainable commentary, solutions, and resources.

Specific to RE-AIM, the outcomes of reach, effectiveness, and adoption demonstrated geographical breadth and a variety of recipient types through each of the implementation strategies. While there is a wide discrepancy between potential LPs and actual LPs, the TTA team leveraged current LP relationships to connect with additional people/organizations interested in developing a TM program. The conversion rate from the number of LPs requesting TTA to those ultimately utilizing that service is very close, which is indicative of a successful strategy. With more than half the people who accessed the Quality Measure Toolkit requesting a PDF of the toolkit, reach and effectiveness were deemed a success. However, there is room for improvement when accounting for the gap between the effectiveness and adoption of the toolkit. Overall, administrators and coordinators tended to participate in the RTTC implementation strategies more often than providers, including the Launch Week, UnConference, TTA, and access of the Toolkit. This could be attributed to time constraints between the two groups and their areas of focus. Administrators may have more time to explore TM opportunities for their organization, while providers may more often be the recipient of TM. Although it is too soon to assess implementation and maintenance, we will look to these domains once the program has had enough time to produce sufficient data.

### Limitations

While all constructs of PRISM were assessed, the RE-AIM domains of implementation and maintenance have not yet been assessed since RTTC implementation is ongoing. This is not a critical limitation; often, all domains of RE-AIM are not assessed, but what is paramount is the value of the information gleaned when using the framework (39). To that end, the domains of reach, effectiveness, and adoption informed outcomes related to the implementation strategies. The fact that maintenance of the implementation strategies has not yet been measured can be considered a limitation. Having a platform such as reflections, however, allows consideration of sustainability from a process perspective instead of an outcomes perspective. Additionally, periodic reflections and implementation science provide tools for replicating successful parts of this national program and avoiding previously noted barriers. This, in turn, supports sustainability. Lastly, the COVID-19 pandemic prevented in-person networking and program promotion, which invariably negatively affected recruitment and participation of LPs, as well as RTTC awareness nationwide. Despite this limitation, LP recruitment occurred with national reach.

### Implications for future work

With the RTTC being a new initiative, there were no preestablished benchmarks on which to build or gauge success. There were, however, lessons learned by using the PRISM and RE-AIM frameworks. Having reflections while implementing the RTTC was helpful. In retrospect, the team could have planned how to use the reflections data to guide operations by tracking the adaptations mentioned during the reflections. With the RTTC team developing the implementation strategies while implementing, future efforts can track specific adaptations mentioned in the reflections to guide the Center’s operations. Nonetheless, the regular group identification of challenges and modifications yielded an overarching view of major changes made within the Center. As the work of the RTTC continues, strategies to recruit and engage LPs will be a priority. One area the RTTC team can address is the time commitment needed by LPs to establish the various TM models. While the value proposition of each model is clearly laid out, documenting specific startup logistics such as staffing time or financial commitment may help LPs better choose a TM model for their workforce.

## Conclusion

This paper demonstrates how PRISM constructs were used to identify contextual factors that influenced and guided RTTC implementation strategies while the RE-AIM domains framed their evaluation. Ongoing reflections allowed the RTTC team to improve implementation team processes in real time. It was helpful to have PRISM and RE-AIM (theory) and reflections (method) to anchor implementation practice and improvement. Teams implementing multifaceted programs may consider adapting the reflections template to prompt discussions of PRISM or other relevant constructs. Moreover, reflections can be used as both documentation and an opportunity for team sensemaking. Similar approaches have been used to tackle complex problems in health care settings (40). Overall, using the PRISM and RE-AIM frameworks allowed the RTTC team to structure a complex, multi-initiative Center with expansive geographical reach into a manageable undertaking. Throughout implementation, it became evident that understanding the context behind programmatic decisions as well as adjusting the implementation process as the program evolved, allowed for a more comprehensive, robust and structured implementation. Such flexibility supported recipients and helped the RTTC reach its goal of becoming a national resource for establishing high-quality telementoring programs that empower the health workforce to improve the health and care of rural Americans.

## Data Availability

The raw data supporting the conclusions of this article will be made available by the authors, without undue reservation.
